# Facet joint degeneration—An initial procedure of the cervical spine degeneration

**DOI:** 10.1002/jsp2.1241

**Published:** 2023-01-02

**Authors:** Qiliang Shang, Dong Wang, Di Wang, Pandi Peng, Han Wang, Haoruo Jia, Jianxin Mao, Chu Gao, Mu Du, Xin He, Yachao Ma, Chao Zheng, Liu Yang, Zhuojing Luo, Xueyu Hu

**Affiliations:** ^1^ Institute of Orthopedic Surgery, Xijing Hospital Fourth Military Medical University Xi'an People's Republic of China; ^2^ Medical Research Institute Northwestern Polytechnical University Xi'an People's Republic of China; ^3^ Pharmacy Department Air Force Hospital of Eastern Theater Command Nanjing People's Republic of China

**Keywords:** degeneration, pain, pre‐clinical models, structure function relationships

## Abstract

**Objective:**

This study aims to emphasize the initiating role of facet joint (FJ) degeneration in the process of cervical spine degeneration induced by tangential load, and we further validate it in a novel cervical spine degeneration animal model.

**Methods:**

The characteristics of cervical degeneration in patients of different ages were summarized through case collection. In the rat models, Hematoxylin–Eosin, Safranin O staining, and micro‐computed tomography were used to show the histopathological changes and bone fiber structure of FJ and the height of intervertebral disc (IVD) space. The ingrowth of nociceptive sensory nerve fibers was observed by immunofluorescence staining.

**Results:**

FJ degeneration without IVDs degeneration was more common in people with cervical spondylosis in young patients. The obvious degeneration phenotypes of the FJs preceded the IVDs at the same cervical segment in our animal model. The SP^+^ and CGRP^+^ sensory nerve fibers were observed in the articular subchondral bone of degenerated FJs and porous endplates of degenerated IVDs.

**Conclusion:**

The FJ degeneration may act as the major contributor to cervical spine degeneration in young people. The dysfunction of functional unit of spine, not a certain part of IVD tissue, results in the occurrence of cervical degeneration and neck pain.

## Graphical abstract

This work reports that under the long‐term tangential load, the facet joint degeneration precedes the intervertebral disc degeneration at the same cervical segment, which may act as the major contributor to cervical spine degeneration in young people. We also established a novel cervical spine degeneration animal model, which can better mimic the cervical spine degeneration of the current young population.

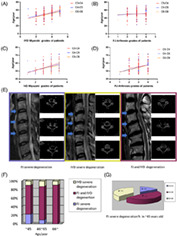

## 1 INTRODUCTION

Cervical degeneration is an age‐related disease that involves degeneration of the intervertebral discs (IVDs), facet joints (FJs), and connective tissue. This disorder was reported to be the major cause of mechanical or axial neck pain, compression, and inflammation of the cervical nerve roots or the adjacent cervical spinal cord.^1^ In a 2015 summary of the worldwide prevalence of neck pain, more than one‐third of people worldwide suffered from mechanical neck pain lasting more than 3 months, which imposed a heavy burden on society and individuals.^2^

It is widely accepted that aging is an important risk factor for cervical degenerative diseases. One population‐based study showed that disc degeneration phenotypes were observed in more than 80% magnetic resonance imaging (MRI) images of people older than 50.^3^ Age‐related disc degeneration with loss of disc height leads to the changes in load‐bearing structure of the spine, which is thought to contribute to the degeneration of other appendages of the spine, such as FJ.^4^ But the occurrence of FJ degeneration (FJD) is not always caused by the process of intervertebral disc degeneration (IDD). The force distribution of IVDs and FJs significantly changed in cervical region due to the different anatomical structure and mechanical loading,^5^ which makes FJs more easily subject to tangential load than IVDs. Therefore, we suspect that the degeneration of the cervical spine may be induced by tangential load on FJs, which is quite different from the viewpoints in previous studies. In order to observe the degeneration characteristics of cervical FJs and IVDs, we collected clinical imaging data to analyze the degeneration characteristics of FJs and IVDs. The cervical spine sustains large compressive loads without damage or instability when the compressive load is tangential to the curve of the cervical spine in the neutral, relaxed posture. However, the compressive loads will produce a large bending moment and tangential forces when the cervical spine is in the state of flexion or extension.^6^ In addition, there are also researches based on finite element model showed that the structure of FJs provided the major stability under tangential loads caused by large flexion and extension.^7^, ^8^ Therefore, we hypothesized that the FJs suffers greater tangential force and leads to degeneration when the cervical spine is in the state of flexion or extension for a long time. To verify this hypothesis, we designed a “pendant” animal model to observe the FJD and IVD at the same segment by applying tangential force to the cervical spine.

It has been reported that the IDD induced by puncture in rat resulted in a significant low back pain phenotype, such as increased mechanical hyperalgesia and thermal hyperalgesia,^9^ which suggests that disc injury may account for part of low back pain. Another study reported that FJ pain accounts for 39%–67% of neck pain, which is much higher than the proportion of low back pain.^10^ However, due to the limitation of effective method to check the source of neck pain in clinical practice, it is still difficult to figure out which part of the cervical spine contributes the major source of neck pain. Therefore, it is of vital importance to establish an appropriate animal model of cervical degeneration and pay more attention to the source of neck pain.

In this study, we first collected 102 MRI and computed tomography (CT) images of patients with cervical spine‐related diseases and analyzed the relationship between FJ and IVD degeneration, and then we established a rat “pendant” model to observe the degeneration of the FJs and IVDs in the same segment.

## 2 MATERIALS AND METHODS

### 2.1 Patient samples

We collected the case data of neck symptomatic cases (include neck pain, shoulder pain, neck stiffness, upper limb pain or numbness) who sought care at Xijing hospital from 2017 to 2021. After excluding cervical spine fractures, tumors, and patients who had received cervical spine surgery, a total of 102 patients (64 males, 38 females, average age = 50.44 ± 10.63) were included in the study (all information of the patients was shown in Table S1). There were 27 cases in the ~45 group, 69 cases in the 46 ~ 65 group and 6 cases in the ~66 group. IDD severity was assessed by three blinded spine surgeons according to the Miyazaki grading system by images of MRI.^11^ MRI was performed using a 3.0T system (GE) to obtain T2‐weighted images (repetition time, 2480 ms; echo time, 104 ms; field of view, 32 mm × 32 mm; slice thickness, 3.5 mm). FJD severity was assessed by the other three blinded spine surgeons according to the Pathria's grading system^12^ according to images of CT. IVDs and FJs of three segments (C3‐C4, C4‐C5, and C5‐C6) were evaluated. Patients with moderate IDD (Grade 1–2) and severe FJD (Grade 3–4) were grouped into FJ severe degeneration group. And patients with moderate FJD (Grade 1–2) and severe IDD (Grade 3–5) were grouped into IVD severe degeneration group. The rest was classified as FJ and IVD degeneration group.

### 2.2 Animal handling

All animal experimental procedures were approved by the Ethics Committee of the Experimental Animal Center of Air Force Military Medical University. Twenty‐four 8‐week‐old male SD rats of SPF grade, weighing about (220 ± 40) g, were provided by the Animal Experiment Center of Air Force Military Medical University. SD rats were randomly selected for inhalation anesthesia with isoflurane at a flow rate of 0.2 ml/min. After successful anesthesia, an 80 g pendant was placed on the neck, to provide a tangential load (Figure S1). Six rats were sacrificed at each time point (0th, 8th, 16th, and 20th weeks), and C2‐C7 cervical vertebrae were removed for follow‐up researches. Con group were these rats hanged pendant after 0 weeks. The pendants worn by animals for the whole duration of the experiment. All rats were reared in the Animal Experiment Center of Air Force Military Medical University with a light–dark cycle of 12 h. The rats in each group were euthanized by inhalation of overdose of isoflurane. C4‐C5 segments were fixed in 4% paraformaldehyde for 24 h for micro‐CT scanning, histological and immunofluorescence analysis.

### 2.3 Micro‐CT measurements

Specimens were fixed in foam blocks and examined by Micro‐CT (Skyscan 1276). A resolution of 10.0 μm per pixel was used to measure the vertebral and FJs. The region of interest (ROI) of FJ was defined as the joint structure within a standard rectangular frame, the size of which was 2.5 mm × 2.5 mm. The center point was located at the midpoint of the left C4‐C5 joint space. The ROI of IVD was defined as the endplate structure, and the thickness was 0.6 mm. The schematic diagram of ROI was show in Figure S2. Images were reconstructed and analyzed by Nrecon v1.6 and CTAn v1.9 (Skyscan US). The bone mineral density (BMD, mg/cm^3^) and bone volume/trabecular volume (BV/TV, %) were measured and the disc height index (DHI) was measured in the midsagittal plane of the C3‐C6 vertebral bodies. DHI was calculated as a previous study described.^13^ Changes of the DHI were expressed as %DHI (%DHI = postoperative DHI/preoperative DHI).

### 2.4 Histopathologic analysis

The cervical vertebrae of rats were fixed in 4% paraformaldehyde for 48 h and then immersed in 10% ethylenediaminetetraacetic acid (pH 7.4) for 4 weeks. The C4/5 segments including the IVD and FJs were separated longitudinally along the pedicle and preserved. Intact IVDs and FJs were separately embedded in paraffin. The sagittal plane of the IVD specimen and the cross‐section of the FJ specimen were cut into 7 μm thick slices for histological staining. Hematoxylin–Eosin (HE) and Safranin O (SO) staining were performed by using respective staining kit according to standard protocols (Solarbio). Histological scores were assessed as described previously.^14^, ^15^

### 2.5 Immunofluorescence staining

Histological sections were incubated with Aggrecan (Millipore, AB1031) (1:100), Calcitonin gene‐related peptide (CGRP, Millipore, C8198) (1:200) or Substance P (SP, Millipore, AB1566) (1:100) at 4°C overnight, followed by secondary antibodies (1:200) at 37°C for 2 h and finally incubated with 4′,6‐diamidino‐2‐phenylindole (DAPI, Beyotime, C1002) for 2 min. Each step was followed by washing with phosphate buffer saline three times for 5 min. The sections were observed under a fluorescence microscope. The ratio of the positive fluorescence area to the ROI area to quantify the fluorescence intensity. ImageJ software was used to quantify the value of fluorescence signals for further quantitative analysis.

### 2.6 Statistical analysis

Data are expressed as the mean ± SD. Single‐factor linear regression was used to analyze the relationship between the age and degeneration grade (IVD or FJ). The chi‐square test was used to compare the composition ratio among groups. Difference among multiple groups were analyzed by one‐way analysis ANOVA of variance followed by Tukey's multiple‐comparison post hoc test and histological scores among multiple groups were analyzed using the Kruskal–Wallis *h*‐test. All statistical analyses were performed using GraphPad Prism software (version 8.0) and SPSS 22.0. Differences were considered significant at *p* < 0.05.

## 3 RESULTS

### 3.1 The degree of FJD is independent of age in young patients with cervical spondylosis

**FIGURE 1:**
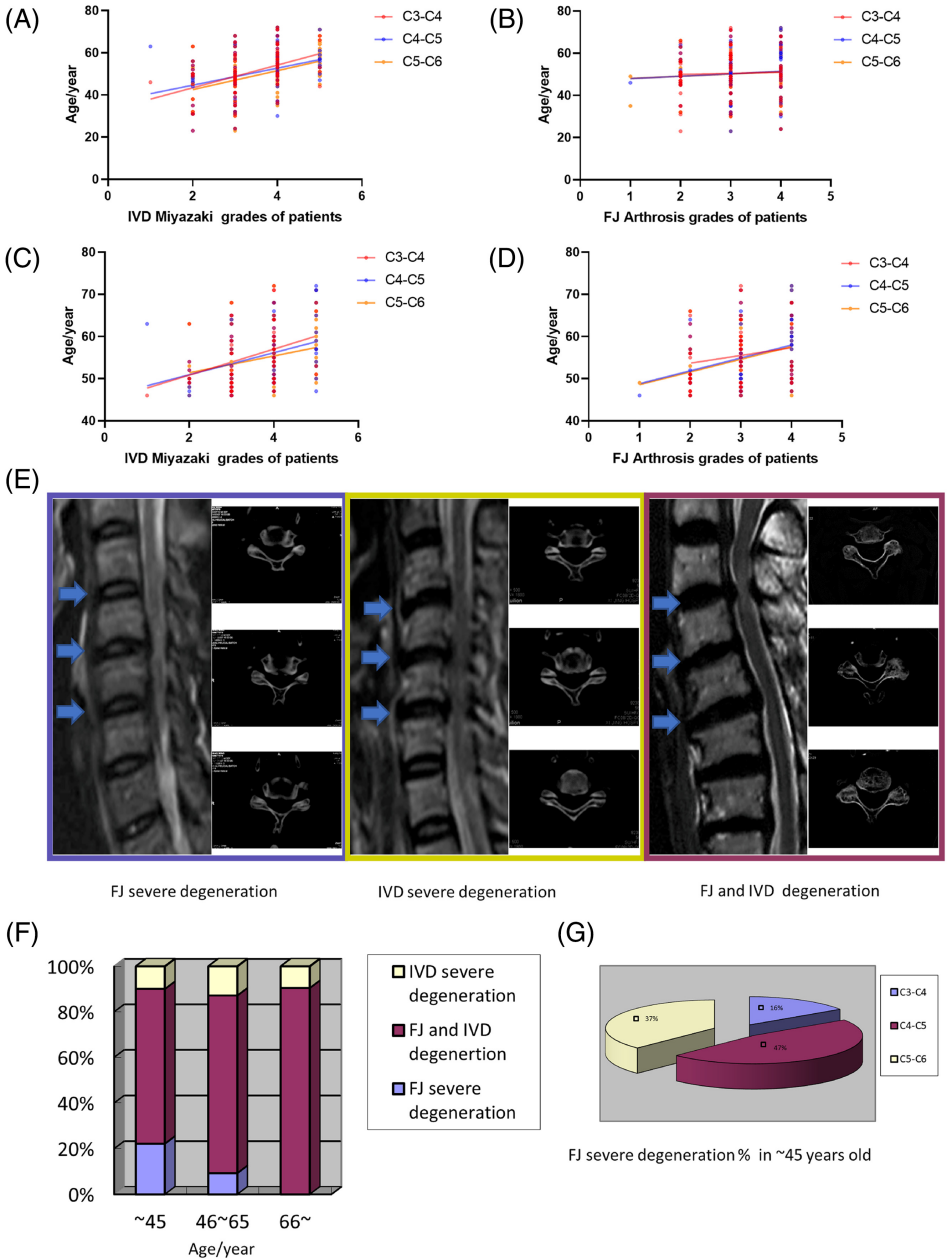
The degree of facet joint degeneration is independent of age in young patients with cervical spondylosis. Single‐factor linear regression analysis of the relationship between the ages and intervertebral disc (IVD) Miyazaki grades (A) or FJ Arthrosis grades (B) based on 102 patients. Single‐factor linear regression analysis of the relationship between the ages and IVD Miyazaki grades (C) or FJ Arthrosis grades (D) based on 75 patients who are older than 45 years old. (E) Representative magnetic resonance imaging (left, IVDs C3‐C6 are marked by blue arrows) and computed tomography (right, FJs C3‐C6) images of FJ severe degeneration (blue box), IVD severe degeneration (yellow box), FJ and IVD degeneration (red box). (F) The distribution of IVD severe degeneration group, FJ severe degeneration group, FJ and IVD degeneration group in different age groups. (G) Prevalence of FJ severe degeneration by different segment. Single‐factor linear regression was used to analyze the relationship between the age and degeneration grade (IVD or FJ). The chi‐square test was used to compare the composition ratio between groups.

Previous studies have reported that age is positively correlated with the severity of IDD.^15^, ^16^, ^17^ In this study, we first analyzed the relationship between age and IDD severity. The Miyazaki grade of IVDs at C3‐C6 levels was positively correlated with age (C3‐C4: *Y* = 5.391 × *X* + 32.63, *R* ^2^ = 0.172, *p* < 0.001; C4‐C5: *Y* = 4.044 × *X* + 36.56, *R* ^2^ = 0.113, *p* = 0.005; C5‐C6: *Y* = 4.501 × *X* + 33.54, *R* ^2^ = 0.168, *p* < 0.001) (Figure 1A), which was consistent with previous studies. However, the severity of FJD did not show a co‐relationship with age (C3‐C4: *Y* = 0.5158 × *X* + 48.94, *R* ^2^ = 0.001, *p* = 0.716; C4‐C5: *Y* = 1.151 × *X* + 46.82, *R* ^2^ = 0.007, *p* = 0.417; C5‐C6: *Y* = 1.072 × *X* + 46.97 *R* ^2^ = 0.006, *p* = 0.451) (Figure 1B). We further analyzed the data and found that some young cases with serious FJD disrupted the linear relationship. With the data from patients younger than 46 years old removed, the results from re‐analysis showed that both the severity of IDD and FJD at the C3‐C6 segment were positively correlated with age (IDD C3‐C4: *Y* = 3.083 × *X* + 44.69, *R* ^2^ = 0.120, *p* = 0.002; C4‐C5: *Y* = 2.615 × *X* + 45.71, *R* ^2^ = 0.124, *p* = 0.002; C5‐C6: *Y* = 1.994 × *X* + 47.42, *R* ^2^ = 0.073, *p* = 0.019) (FJD C4‐C5: *Y* = 3.059 × *X* + 45.75, *R* ^2^ = 0.112, *p* = 0.003; C5‐C6: *Y* = 3.015 × *X* + 45.56, *R* ^2^ = 0.092, *p* = 0.008) (Figure 1C,D). These results indicate that age is not a major contributing factor in FJD in some young groups. Moreover, we divided all the patients into three groups according to the severity of IDD and FJD (IVD severe degeneration group, FJ severe degeneration group, FJ and IVD degeneration group). Representative images were showed in Figure 1E. We also analyzed their distribution in different age groups. The results showed that the proportion of FJ severe degeneration in ages ~45 group was higher than that in the other two groups, and the severe FJD was mainly distributed in the C4‐C5 and C5‐C6 segments (Figure 1F,G).

### 3.2 Significant FJD phenotypes are observed in a new rat pendant model

**FIGURE 2:**
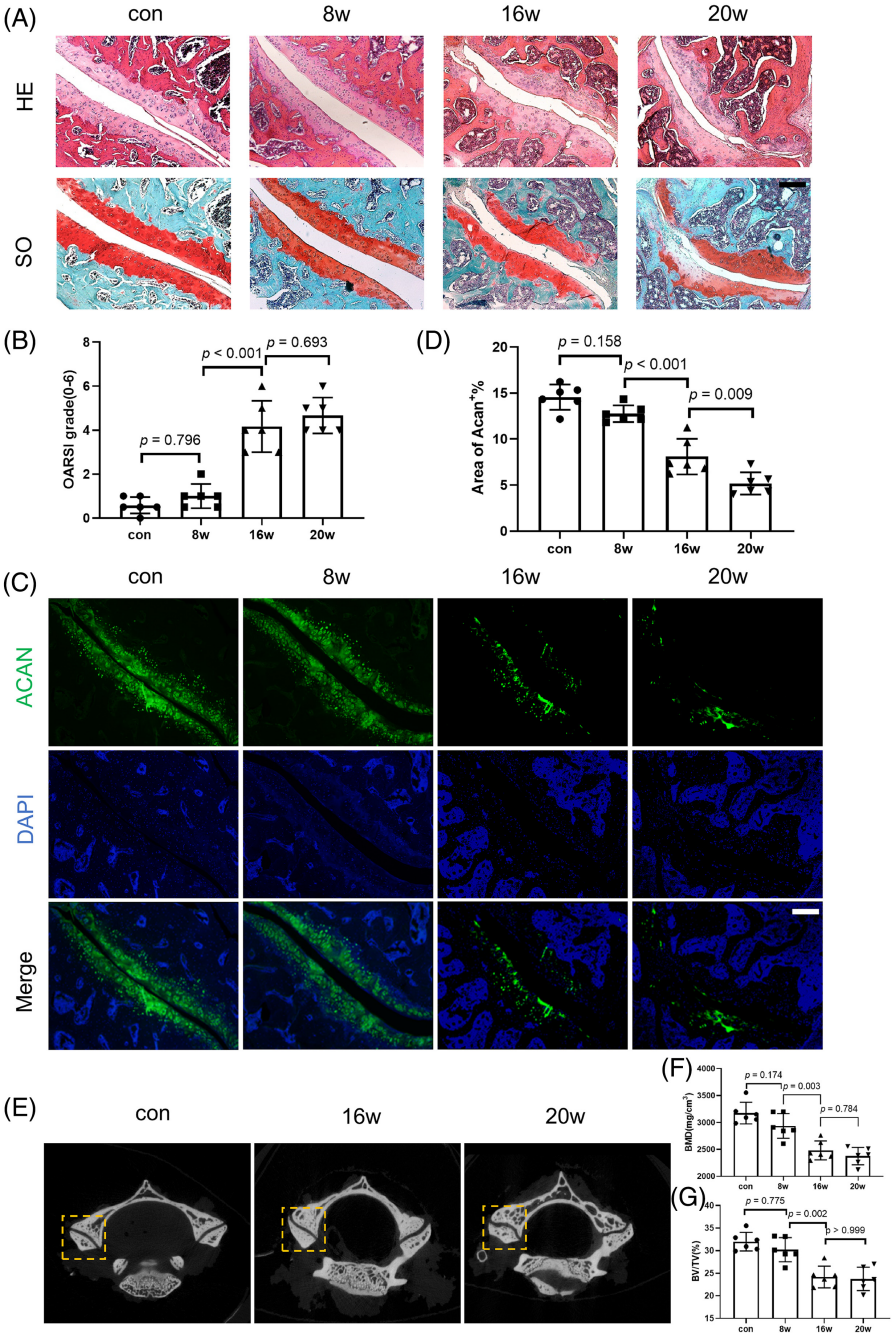
Significant facet joint degeneration phenotypes are observed in a new rat pendant model. (A) Hematoxylin–Eosin and Safranin O staining of C4/5 facet joints (FJs) at 0, 8, 16, 20 weeks after pendant hanging. (B) Histological score of (A). (C) C4/5 FJs representative images of immunofluorescence of Aggrecan at 0, 8, 16, 20 weeks after pendant hanging. (D) Percentage of Aggrecan+ area of (C), The region of interest is the whole region of FJs in the last line of (C). (E) Representative micro‐computed tomography (CT) scans of the FJs. Quantitative analysis of bone mineral density (F) and bone volume/trabecular volume (G) in FJs determined by micro‐CT. *n* = 6. Scale = 200 μm. The data are presented as the means ± SDs. The significant differences of Aggrecan+ area was determined by one‐way ANOVA with Tukey's multiple‐comparison post hoc test. Histological scores among multiple groups were analyzed using the Kruskal–Wallis *h*‐test.

In order to observe the changes of cervical FJs under the tangential force, we hung an 80 g “pendant” (about 1/3 of the rat's weight) on the neck of rats (Figure S1A,B). Histological analysis using HE and SO staining showed some significant changes in the structure of FJs. In the 8w group, only some chondrocytes cells were sparsely arranged in the C4‐5 FJs (ipsilateral, left) and histopathological scores showed no statistical difference as well when compared with the control group. In the 16w group, the FJ showed some degenerative phenotypes, such as some irregularity on articular surface, cytoplasmic vacuolation in chondrocytes cells and cartilaginus ossification on articular margin, and the histopathological scores was significantly higher when compared with the control group and the 8w group. In the 20w group, more severe degenerative phenotypes were observed, such as disappeared articular cartilage, emerging osteophytes, and chondrocyte clustering phenomenon. The histopathological score was also significantly higher than that in the 8w group, but it showed no significant difference from the 16w group (Figure 2A,B). Images of immunofluorescence staining showed that the expression of aggrecan in the FJ cartilage significantly decreased at 16w group and 20w group (Figure 2C,D). Micro‐CT imaging showed that the less smooth articular surface of the FJ was initially observed at 16w, and osteophyte formation started to be evident at 20w (Figure 2E). Bone mass analysis showed that BMD and BV/TV were significantly decreased at 16w and 20w (Figure 2F). These results indicate that tangential loading is a major risk factor for FJD and significant FJD phenotypes were initially observed in our new rat pendant models 16w after operation.

### 3.3 Significant IDD phenotypes lag behind the FJD phenotypes at the same segment in rat pendant models

**FIGURE 3:**
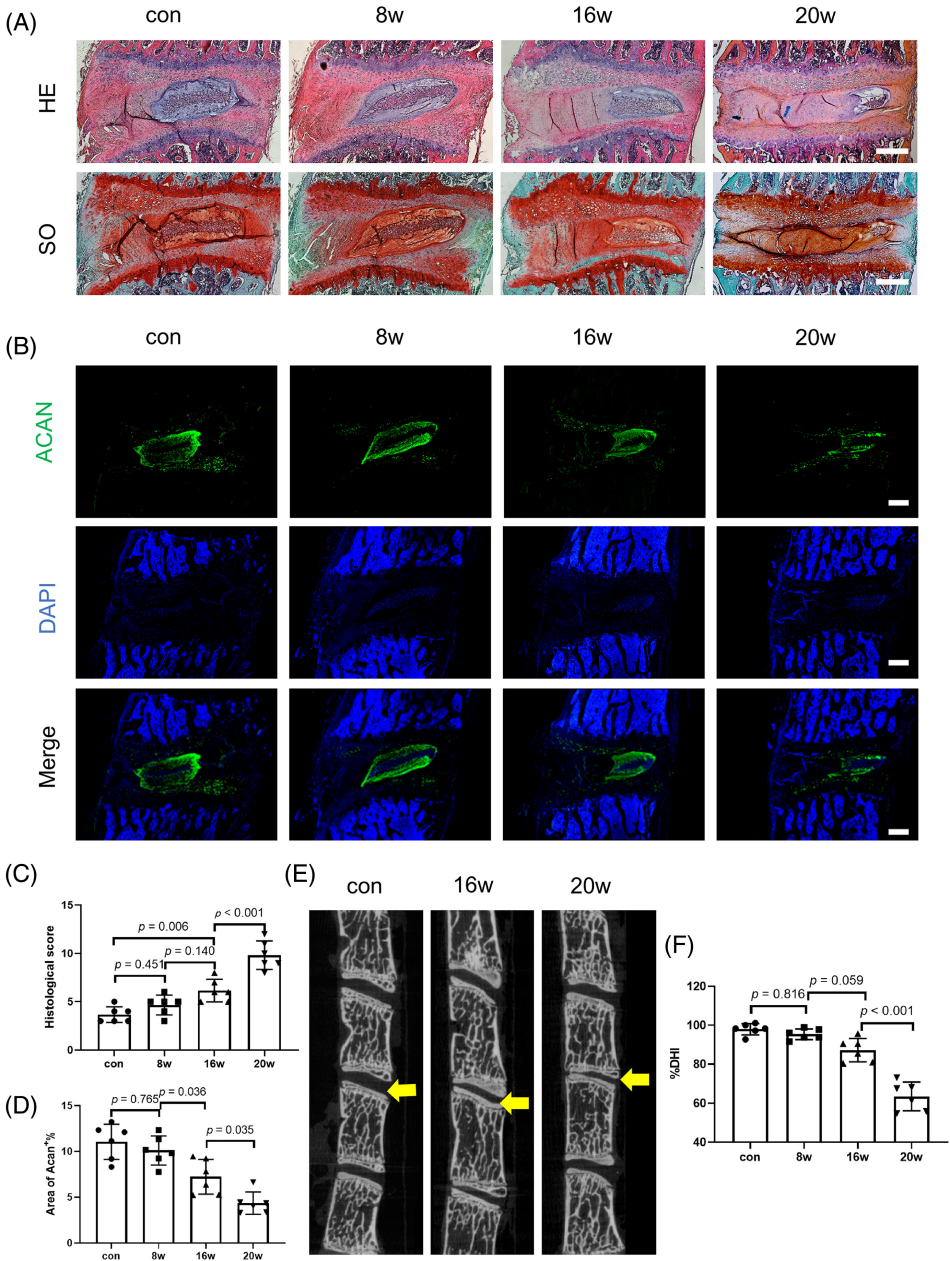
Significant intervertebral disc degeneration phenotypes lag behind the facet joint degeneration phenotypes at the same segment in rat pendant models. (A) Hematoxylin–Eosin and Safranin O staining of C4/5 intervertebral discs (IVDs) at 0, 8, 16, 20 weeks after pendant hanging. (B) C4/5 IVDs representative images of immunofluorescence of Aggrecan at 0, 8, 16, 20 weeks after pendant hanging. (C) Histological score of (A). (D) Percentage of Aggrecan+ area of (B), the region of interest is the whole region of IVDs in the last line of (B). (E) Representative micro‐computed tomography (CT) scans of the IVD. Yellow triangles: cervical IVDs of C4‐C5. (F) DHI% of micro‐CT. *n* = 6. Scale = 200 μm. The data are presented as the means ± SDs. The significant differences of Aggrecan+ area was determined by one‐way ANOVA with Tukey's multiple‐comparison post hoc test. Histological scores among multiple groups were analyzed using the Kruskal–Wallis *h*‐test.

In order to explore the effect of tangential force on cervical IVDs, we also observed the histological changes of cervical IVDs in rat pendant models. Histological analysis using HE and SO staining showed lagging degenerative phenotypes of cervical IVDs when compared with the cervical FJs. Compared to the control group, no degenerative changes were observed in the cervical IVDs in the 8w group and only some minor changes (slightly squeezed structure and reduced cell number of nucleus pulposus) were observed in the 16w group. In the 20w group, some striking changes of IVDs were observed, such as the obviously squeezed nucleus pulposus, the disorganized arrangement of the annulus fibrosus, the dramatically reduced cell numbers and significantly degraded extracellular matrix, and histopathological scores also reflected these changes (Figure 3A,B). Immunofluorescence staining showed that the expression of aggrecan in the nucleus pulposus slightly decreased in the 16w group and significantly decreased in the 20w group (Figure 3C,D). Micro‐CT imaging showed that the C4‐C5 intervertebral space was lost in the 20w group and the %DHI was significantly lower than that of other groups (Figure 3E,F). The BMD of the endplates were decreased significantly at 20 week, and no significant changes were observed in BV/TV (Figure S3). These results indicate that under the tangential force, the occurrence of cervical FJD is earlier than that of IDD at the same cervical segment.

### 3.4 Nociceptive sensory nerve fibers grow into the subchondral bone of FJs and the endplate of IVDs

**FIGURE 4:**
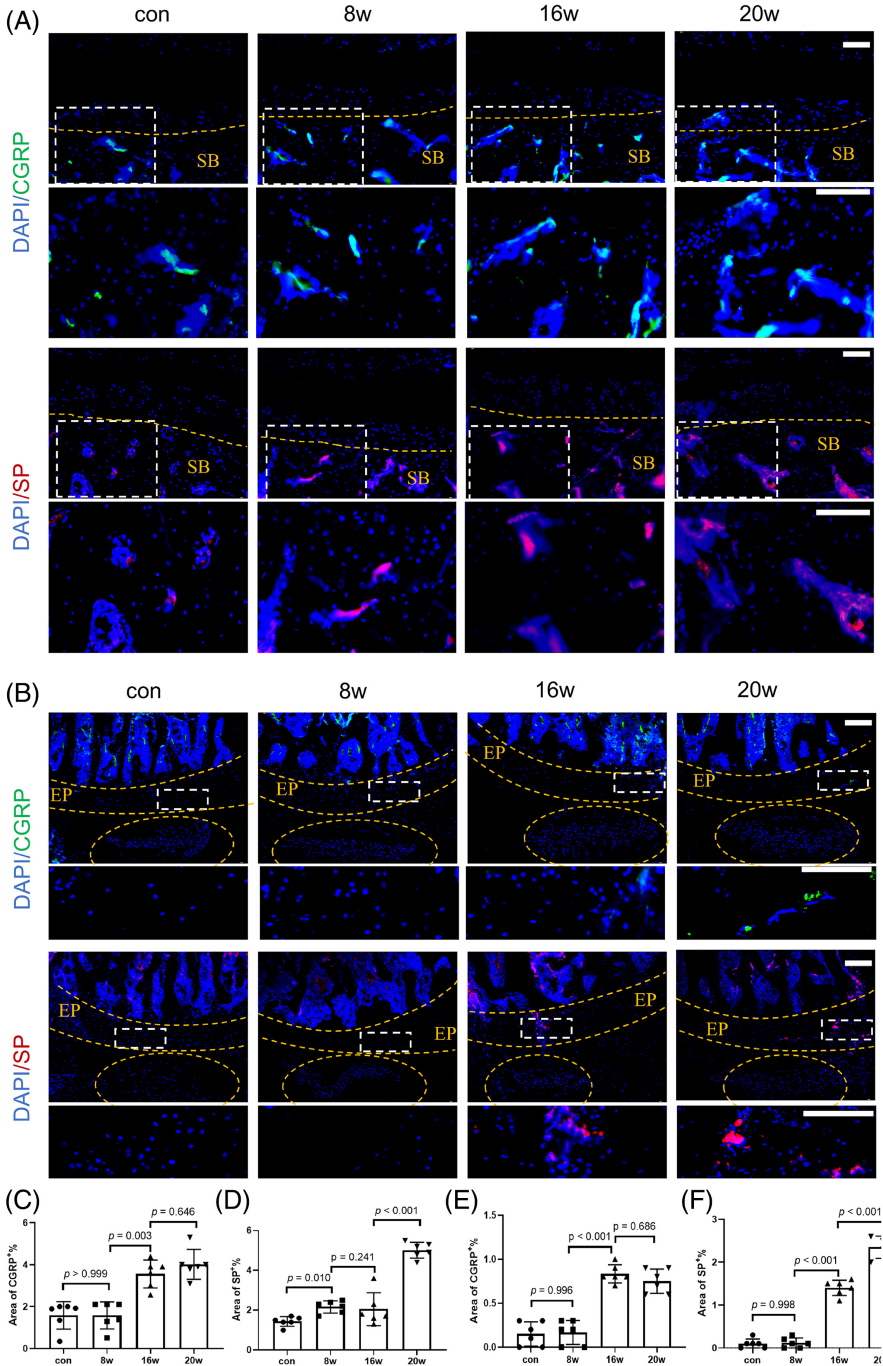
Nociceptive sensory nerve fibers grow into the subchondral bone of facet joints (FJs) and the endplate of intervertebral discs (IVDs). (A) Immunofluorescence analysis of calcitonin gene‐related peptide (CGRP^+^) sensory nerve fibers (first row, green) and SP^+^ sensory nerve fibers (third row, red) in rat cervical FJ subchondral bone of C4/5 at different time points. (B) Immunofluorescence analysis of CGRP^+^ sensory nerve fibers (first row, green) and SP^+^ sensory nerve fibers (third row, red) in rat cervical caudal endplates of C4/5 at different time points. The second and fourth rows are enlarged images of the white boxes in the first and third rows. (C and D) Percentage of CGRP^+^ area and SP^+^ area of (A), the region of interest (ROI) is the subchondral bone of FJs defined by the yellow curves in (A). (E and F) Percentage of CGRP^+^ area and SP^+^ area of (B), the ROI is the endplate of IVDs defined by the yellow curves in (B). *n* = 6. Scale = 50 μm. The data are presented as the means ± SDs. The significant differences among the five groups were determined by one‐way ANOVA with Tukey's multiple‐comparison post hoc test.

In order to explore whether FJ‐derived or disc‐derived neck pain occurs under the tangential force, we detected the distribution of SP^+^ nerve fibers and CGRP^+^ nerve fibers in FJs and IVDs. Immunofluorescence staining showed that SP^+^ nerve fibers in the subchondral bone of FJs were initially observed in the 8w group, and the number of CGRP^+^ nerve fibers in the 16w and 20w groups was significantly higher than that in the control and 8w groups (Figure 4A,C,D). Moreover, the number of SP^+^ and CGRP^+^ nerve fibers significantly increased at 16w and 20w in the porous endplate of IVDs, either (Figure 4B,E,F). These results suggest that in cervical degenerative diseases, both IVDs and FJs are the source of pain.

## 4 DISCUSSION

The IVD tissue is an important load‐bearing structure of the spine, and its degenerative change is considered a major cause of neck pain and low back pain.^18^, ^19^, ^20^ In previous studies, the degeneration of IVDs was considered an initiating factor for the degeneration of the spine, while the degeneration of FJs acted as an accompanied factor.^21^, ^22^ However, these studies only focused on the degeneration of lumbar spine. Compared to the lumbar spine, the anatomical structures of cervical spine show some significant differences, such as smaller discs, increased relative FJ surface areas (FJ surface areas/endplate area) and changed orientation of FJ surface.^23^ These anatomical changes lead to different stress distribution of FJs and IVDs in the lumbar spine and cervical spine. From our clinical data, we found that the FJD in some young patients was nonage related, and severe FJD and slight IDD at the same segment were observed. In combination with the social background of young people who have been computer working or mobile phones playing for a long time, we speculated that our clinical results may be related to the increased tangential force of these cervical spines as a result of prolonged non‐neutral positioning. We will collect more comprehensive information, such as mobile phone use time, patients' work types and working hours, in our subsequent research to avoid the limitation that our clinical data only provides indirect evidences.

However, it is still unclear whether tangential load is great enough to cause FJ degeneration and whether FJD is prior to IDD at the same segment under tangential force. Lack of appropriate animal model is a major reason. In 1991, Shimpei Miyamoto^24^ established a cervical instability model by excised the cervical spinous process, interspinous ligament, supraspinous ligament, and paravertebral muscles in mice and some degenerative phenotypes were observed 24 weeks after surgery. Subsequently, a series of cervical degeneration models were explored to shorten the modeling time by removing more neck muscles or even FJs.^25^, ^26^, ^27^, ^28^ However, instead of mimicking abnormal external forces, these models altered the internal biomechanical properties of the spine, which makes the evidence offered by these models rather weak. Additionally, most animal models of lumber FJ osteoarthritis (LFJ OA) are reported on the lumbar spine. Kai Gong et al.^29^ established a rat FJ OA model using monosodium iodoacetate injection. Progressive cartilage degeneration and pain hypersensitivity were observed. Miao Li et al.^30^ placed the mice in a beaker containing limited water to induce the bipedal standing posture and the LFJ OA was observed at 24 weeks. Due to the differences in anatomy, as described previously, the conclusions of these models do not apply to the cervical spine, and changes in the IVDs of the same segment have not been observed. The results of our preliminary experiment verified that the weight of 80 g did not affect the feeding, drinking and daily activities of the rats. In addition, sponge tape on the contact surface between the pendant and the neck skin effectively prevent the occurrence of skin abrasion. The pendant was placed between the C4‐C5 spinous processes according to the previous experimental observation.

Although we confirmed that under the tangential force, the occurrence of cervical FJD was earlier than that of IDD at the same segment by this “pendants” model, no direct evidence showed which event was the exact source of neck pain. It has been reported that nerve ingrowth and secretion of pain‐related factors are two common phenotypes of cervical degeneration. The two pain‐related phenotypes have been used as evaluation indicators in cartilage endplate tissue and subchondral bone.^31^, ^32^, ^33^ With tissue damaged, DRG neurons are highly sensitive due to the activation of nociceptors by various neurotransmitters, including SP and CGRP, released by nociceptive nerve fibers, resulting in hyperalgesia. Thus, we detected the ingrowth of SP^+^ and CGRP^+^ sensory never fibers in the subchondral bone of C4/5 FJs and the endplate of IVDs in the same segment. Our results showed the increased ingrowth of SP^+^ and CGRP^+^ sensory nerve fibers in both subchondral bone of C4/5 FJs and the endplate of IVDs at 16th week, indicating combined sources of neck pain. Our study supports an important theory that the dysfunction of functional unit of spine, not a certain part of IVD tissue, results in the occurrence of cervical degeneration and neck pain.^34^, ^35^

Whiplash injury, a special kind of neck injury caused by a sudden load of tangential force (acceleration or deceleration of the head accompanied by hyperextension or flexion of the neck), was reported to cause structural damages to FJs and thus induce FJ pain, which accounted for 54% to 60% of chronic neck pain after whiplash injury.^36^, ^37^, ^38^ Some injures caused by accumulated tangential stress, which are commonly found in people playing mobile phones, computer working, or driving a car or truck for a long time, can also induce a chronic neck pain. A systematic review with meta‐analysis indicated that more than 4 h of computer working time will increase the risk of neck pain.^39^, ^40^ Another study on bus drivers found that the highest percentage of musculoskeletal disorders was discomfort on the neck, which may be related to the frequent steering, gears shifting and braking required by bus drivers.^41^ We boldly define this kind of injury caused by long‐term tangential load as “chronic whiplash injury,” and our rat “pendant” model successfully mimics the chronic injury caused by these lifestyles and provides an effective and stable research model for further study. However, there are some limitations in our model. First, the force loading method is different from the real situation. We use the tangential force of the neck to replace the abnormal force when the head is stretched forward or tilted backward, which weakens the authenticity of the mode. In addition, our model emphasizes the role of tangential force in the process of cervical spine degeneration, and the phenotype may be slightly inconsistent with the cervical degenerative process under comprehensive stress. Our future research will also consider comprehensive stress to explore more appropriate animal models of cervical spine degeneration.

To sum up, our study found that FJs are more easily subjected to tangential stress than IVDs in the cervical segment and FJD may be an initial process of the cervical spine degeneration. In addition, our results also confirmed that chronic neck pain originated from both FJs and IVDs. Finally, we established a novel rat cervical spine degeneration model, which provided an efficient and stable research tools for the study of cervical spine degeneration.

## AUTHOR CONTRIBUTIONS

Qiliang Shang, Liu Yang, and Zhuojing Luo designed the experiments. Qiliang Shang, Dong Wang, Di Wang, Pandi Peng, Chu Gao, Jianxin Mao, and Mu Du carried out most of the experiments. Dong Wang, Yachao Ma, Di Wang, Chao Zheng, and Xueyu Hu helped collecting the samples. Han Wang, Haoruo Jia, and Xin He proofread the manuscript. Qiliang Shang, Dong Wang, and Xueyu Hu analyzed the data and wrote the manuscript. All authors contributed to the article and approved the submitted version.

## CONFLICT OF INTEREST

All the authors declare that without any business or financial relationship that may be construed as potential conflict of interest.

## Supporting information


**Data S1.** Supporting Information.
