# Supplementary material for: Facet joint degeneration—An initial procedure of the cervical spine degeneration
Source: JOR Spine. 2023 Jan 2;6(1):e1241. doi: 10.1002/jsp2.1241 (PMC10041371; doi:10.1002/jsp2.1241)
Supplement: Supplementary file 1 — Data S1. Supporting Information. [file JSP2-6-e1241-s001.doc]

**Supplementary Fig S1.**


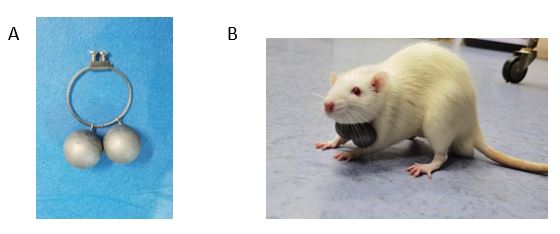


（A）Picture of pendent. (B) The state of the rat after hanging pendent on the neck.

**Supplementary Fig S2.**

**
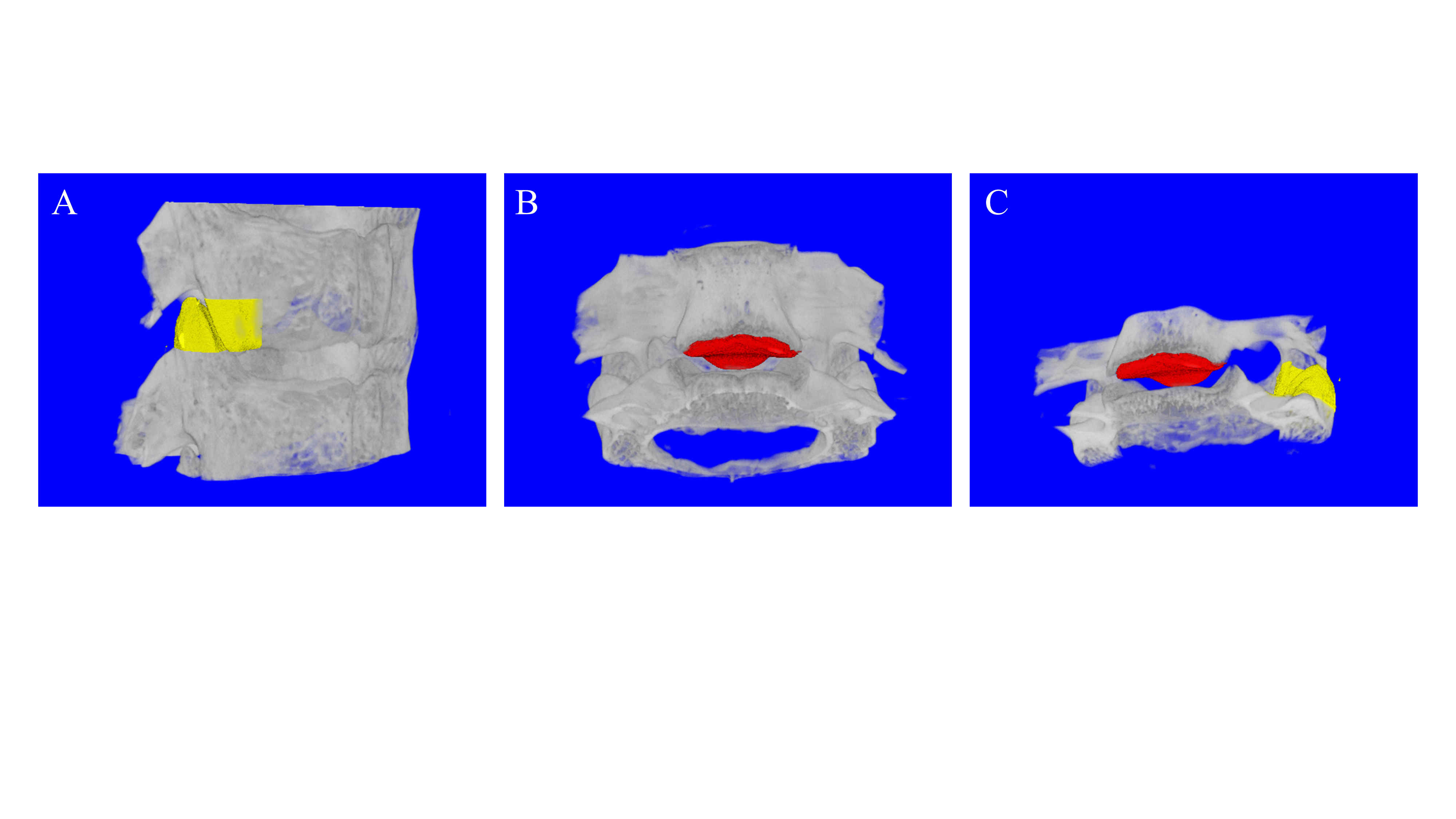
**

The schematic diagram of ROI. (A)Facet joint is marked in yellow. (B) Endplate is marked in red. (C) ROIs are displayed in the same spinal segment.

**Supplementary Fig S3.**

**
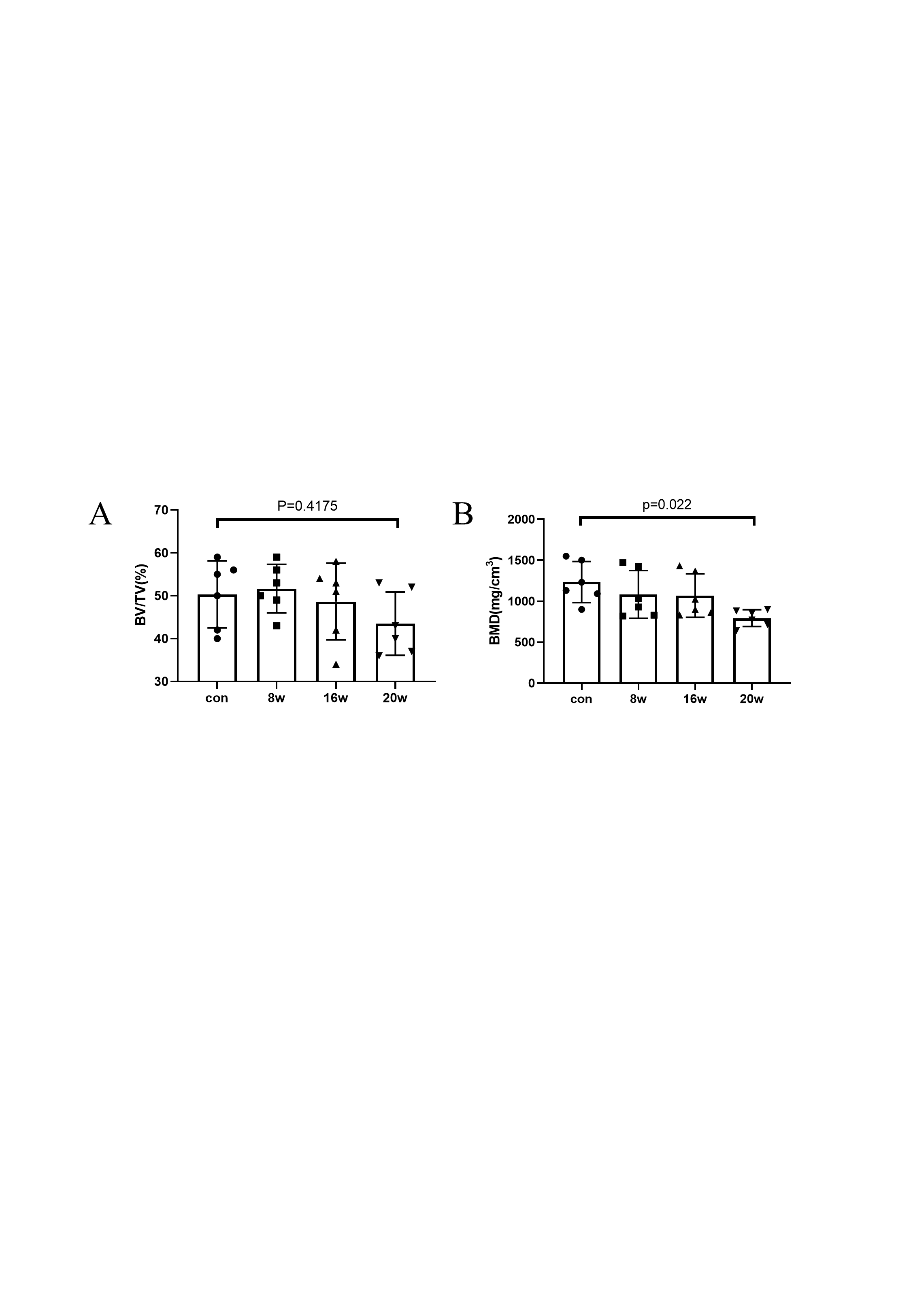
**

Quantitative analysis of bone volume/trabecular volume (BV/TV) (A) and bone mineral density (BMD) (B) in endplates determined by μCT.

**Supplementary table S1**

Demographic data of patients

| Patient No. | Gender | Age | Levels | Miyazaki grading (IVD) | Arthrosis grading (FJ) |
| --- | --- | --- | --- | --- | --- |
| 1 | M | 50 | C3-C4 | 3 | 2 |
|  |  |  | C4-C5 | 4 | 3 |
|  |  |  | C5-C6 | 3 | 4 |
| 2 | M | 47 | C3-C4 | 3 | 3 |
|  |  |  | C4-C5 | 2 | 4 |
|  |  |  | C5-C6 | 3 | 3 |
| 3 | F | 51 | C3-C4 | 3 | 2 |
|  |  |  | C4-C5 | 3 | 3 |
|  |  |  | C5-C6 | 3 | 3 |
| 4 | M | 58 | C3-C4 | 3 | 3 |
|  |  |  | C4-C5 | 4 | 4 |
|  |  |  | C5-C6 | 5 | 4 |
| 5 | F | 47 | C3-C4 | 3 | 2 |
|  |  |  | C4-C5 | 3 | 3 |
|  |  |  | C5-C6 | 4 | 3 |
| 6 | F | 47 | C3-C4 | 4 | 4 |
|  |  |  | C4-C5 | 4 | 3 |
|  |  |  | C5-C6 | 4 | 4 |
| 7 | M | 45 | C3-C4 | 3 | 2 |
|  |  |  | C4-C5 | 3 | 2 |
|  |  |  | C5-C6 | 3 | 3 |
| 8 | F | 66 | C3-C4 | 5 | 2 |
|  |  |  | C4-C5 | 4 | 3 |
|  |  |  | C5-C6 | 5 | 2 |
| 9 | F | 56 | C3-C4 | 4 | 2 |
|  |  |  | C4-C5 | 5 | 3 |
|  |  |  | C5-C6 | 3 | 2 |
| 10 | M | 65 | C3-C4 | 3 | 2 |
|  |  |  | C4-C5 | 3 | 4 |
|  |  |  | C5-C6 | 4 | 3 |
| 11 | M | 57 | C3-C4 | 4 | 2 |
|  |  |  | C4-C5 | 5 | 2 |
|  |  |  | C5-C6 | 3 | 2 |
| 12 | M | 41 | C3-C4 | 3 | 3 |
|  |  |  | C4-C5 | 3 | 3 |
|  |  |  | C5-C6 | 4 | 3 |
| 13 | F | 36 | C3-C4 | 3 | 3 |
|  |  |  | C4-C5 | 3 | 4 |
|  |  |  | C5-C6 | 3 | 4 |
| 14 | F | 24 | C3-C4 | 4 | 4 |
|  |  |  | C4-C5 | 4 | 4 |
|  |  |  | C5-C6 | 4 | 4 |
| 15 | F | 64 | C3-C4 | 3 | 3 |
|  |  |  | C4-C5 | 4 | 4 |
|  |  |  | C5-C6 | 4 | 3 |
| 16 | M | 60 | C3-C4 | 3 | 2 |
|  |  |  | C4-C5 | 4 | 2 |
|  |  |  | C5-C6 | 5 | 3 |
| 17 | F | 49 | C3-C4 | 3 | 3 |
|  |  |  | C4-C5 | 4 | 2 |
|  |  |  | C5-C6 | 5 | 3 |
| 18 | F | 48 | C3-C4 | 3 | 3 |
|  |  |  | C4-C5 | 2 | 3 |
|  |  |  | C5-C6 | 2 | 3 |
| 19 | M | 68 | C3-C4 | 4 | 4 |
|  |  |  | C4-C5 | 4 | 4 |
|  |  |  | C5-C6 | 5 | 4 |
| 20 | M | 47 | C3-C4 | 4 | 2 |
|  |  |  | C4-C5 | 5 | 3 |
|  |  |  | C5-C6 | 4 | 4 |
| 21 | M | 50 | C3-C4 | 2 | 2 |
|  |  |  | C4-C5 | 3 | 4 |
|  |  |  | C5-C6 | 2 | 3 |
| 22 | F | 55 | C3-C4 | 4 | 3 |
|  |  |  | C4-C5 | 4 | 3 |
|  |  |  | C5-C6 | 4 | 3 |
| 23 | M | 59 | C3-C4 | 3 | 3 |
|  |  |  | C4-C5 | 3 | 3 |
|  |  |  | C5-C6 | 4 | 3 |
| 24 | M | 49 | C3-C4 | 2 | 2 |
|  |  |  | C4-C5 | 2 | 2 |
|  |  |  | C5-C6 | 3 | 2 |
| 25 | F | 65 | C3-C4 | 4 | 4 |
|  |  |  | C4-C5 | 4 | 4 |
|  |  |  | C5-C6 | 5 | 4 |
| 26 | M | 31 | C3-C4 | 2 | 3 |
|  |  |  | C4-C5 | 2 | 3 |
|  |  |  | C5-C6 | 3 | 3 |
| 27 | M | 57 | C3-C4 | 3 | 3 |
|  |  |  | C4-C5 | 4 | 3 |
|  |  |  | C5-C6 | 5 | 3 |
| 28 | F | 53 | C3-C4 | 4 | 3 |
|  |  |  | C4-C5 | 4 | 4 |
|  |  |  | C5-C6 | 5 | 2 |
| 29 | M | 63 | C3-C4 | 3 | 2 |
|  |  |  | C4-C5 | 3 | 2 |
|  |  |  | C5-C6 | 3 | 2 |
| 30 | M | 68 | C3-C4 | 5 | 3 |
|  |  |  | C4-C5 | 4 | 4 |
|  |  |  | C5-C6 | 4 | 4 |
| 31 | M | 35 | C3-C4 | 3 | 4 |
|  |  |  | C4-C5 | 3 | 3 |
|  |  |  | C5-C6 | 3 | 4 |
| 32 | M | 54 | C3-C4 | 4 | 4 |
|  |  |  | C4-C5 | 4 | 4 |
|  |  |  | C5-C6 | 4 | 4 |
| 33 | M | 46 | C3-C4 | 3 | 2 |
|  |  |  | C4-C5 | 3 | 2 |
|  |  |  | C5-C6 | 4 | 2 |
| 34 | F | 48 | C3-C4 | 3 | 3 |
|  |  |  | C4-C5 | 3 | 3 |
|  |  |  | C5-C6 | 4 | 4 |
| 35 | M | 57 | C3-C4 | 4 | 3 |
|  |  |  | C4-C5 | 3 | 4 |
|  |  |  | C5-C6 | 3 | 3 |
| 36 | F | 59 | C3-C4 | 3 | 3 |
|  |  |  | C4-C5 | 3 | 4 |
|  |  |  | C5-C6 | 4 | 3 |
| 37 | F | 45 | C3-C4 | 3 | 4 |
|  |  |  | C4-C5 | 3 | 3 |
|  |  |  | C5-C6 | 4 | 2 |
| 38 | M | 50 | C3-C4 | 4 | 4 |
|  |  |  | C4-C5 | 5 | 2 |
|  |  |  | C5-C6 | 5 | 3 |
| 39 | M | 51 | C3-C4 | 5 | 4 |
|  |  |  | C4-C5 | 4 | 4 |
|  |  |  | C5-C6 | 5 | 3 |
| 40 | M | 45 | C3-C4 | 4 | 2 |
|  |  |  | C4-C5 | 3 | 2 |
|  |  |  | C5-C6 | 4 | 3 |
| 41 | F | 55 | C3-C4 | 4 | 2 |
|  |  |  | C4-C5 | 4 | 3 |
|  |  |  | C5-C6 | 5 | 3 |
| 42 | F | 49 | C3-C4 | 4 | 3 |
|  |  |  | C4-C5 | 4 | 3 |
|  |  |  | C5-C6 | 5 | 4 |
| 43 | M | 47 | C3-C4 | 3 | 3 |
|  |  |  | C4-C5 | 3 | 3 |
|  |  |  | C5-C6 | 3 | 4 |
| 44 | M | 57 | C3-C4 | 4 | 3 |
|  |  |  | C4-C5 | 5 | 3 |
|  |  |  | C5-C6 | 4 | 3 |
| 45 | M | 50 | C3-C4 | 4 | 4 |
|  |  |  | C4-C5 | 4 | 4 |
|  |  |  | C5-C6 | 4 | 4 |
| 46 | M | 44 | C3-C4 | 5 | 3 |
|  |  |  | C4-C5 | 4 | 4 |
|  |  |  | C5-C6 | 4 | 3 |
| 47 | M | 31 | C3-C4 | 3 | 4 |
|  |  |  | C4-C5 | 3 | 4 |
|  |  |  | C5-C6 | 3 | 4 |
| 48 | M | 32 | C3-C4 | 3 | 3 |
|  |  |  | C4-C5 | 3 | 3 |
|  |  |  | C5-C6 | 3 | 4 |
| 49 | F | 52 | C3-C4 | 3 | 3 |
|  |  |  | C4-C5 | 4 | 2 |
|  |  |  | C5-C6 | 4 | 3 |
| 50 | M | 63 | C3-C4 | 2 | 4 |
|  |  |  | C4-C5 | 1 | 3 |
|  |  |  | C5-C6 | 2 | 4 |
| 51 | M | 68 | C3-C4 | 3 | 4 |
|  |  |  | C4-C5 | 3 | 3 |
|  |  |  | C5-C6 | 3 | 4 |
| 52 | F | 53 | C3-C4 | 5 | 4 |
|  |  |  | C4-C5 | 4 | 3 |
|  |  |  | C5-C6 | 4 | 4 |
| 53 | F | 58 | C3-C4 | 4 | 3 |
|  |  |  | C4-C5 | 4 | 4 |
|  |  |  | C5-C6 | 4 | 3 |
| 54 | F | 44 | C3-C4 | 3 | 4 |
|  |  |  | C4-C5 | 2 | 4 |
|  |  |  | C5-C6 | 3 | 4 |
| 55 | F | 51 | C3-C4 | 4 | 2 |
|  |  |  | C4-C5 | 4 | 2 |
|  |  |  | C5-C6 | 3 | 3 |
| 56 | M | 49 | C3-C4 | 3 | 2 |
|  |  |  | C4-C5 | 4 | 2 |
|  |  |  | C5-C6 | 3 | 1 |
| 57 | M | 38 | C3-C4 | 3 | 4 |
|  |  |  | C4-C5 | 3 | 4 |
|  |  |  | C5-C6 | 4 | 4 |
| 58 | M | 35 | C3-C4 | 3 | 2 |
|  |  |  | C4-C5 | 3 | 2 |
|  |  |  | C5-C6 | 4 | 1 |
| 59 | M | 51 | C3-C4 | 3 | 2 |
|  |  |  | C4-C5 | 3 | 2 |
|  |  |  | C5-C6 | 4 | 3 |
| 60 | M | 36 | C3-C4 | 4 | 3 |
|  |  |  | C4-C5 | 4 | 3 |
|  |  |  | C5-C6 | 4 | 4 |
| 61 | M | 46 | C3-C4 | 3 | 2 |
|  |  |  | C4-C5 | 3 | 1 |
|  |  |  | C5-C6 | 4 | 2 |
| 62 | F | 50 | C3-C4 | 4 | 2 |
|  |  |  | C4-C5 | 4 | 3 |
|  |  |  | C5-C6 | 5 | 3 |
| 63 | F | 30 | C3-C4 | 3 | 3 |
|  |  |  | C4-C5 | 4 | 4 |
|  |  |  | C5-C6 | 3 | 3 |
| 64 | M | 60 | C3-C4 | 4 | 3 |
|  |  |  | C4-C5 | 4 | 4 |
|  |  |  | C5-C6 | 5 | 4 |
| 65 | M | 54 | C3-C4 | 4 | 3 |
|  |  |  | C4-C5 | 5 | 3 |
|  |  |  | C5-C6 | 5 | 4 |
| 66 | M | 61 | C3-C4 | 4 | 3 |
|  |  |  | C4-C5 | 5 | 4 |
|  |  |  | C5-C6 | 5 | 4 |
| 67 | F | 53 | C3-C4 | 4 | 3 |
|  |  |  | C4-C5 | 5 | 4 |
|  |  |  | C5-C6 | 5 | 4 |
| 68 | F | 64 | C3-C4 | 4 | 3 |
|  |  |  | C4-C5 | 4 | 4 |
|  |  |  | C5-C6 | 5 | 4 |
| 69 | M | 45 | C3-C4 | 4 | 2 |
|  |  |  | C4-C5 | 4 | 3 |
|  |  |  | C5-C6 | 5 | 3 |
| 70 | M | 49 | C3-C4 | 3 | 3 |
|  |  |  | C4-C5 | 3 | 2 |
|  |  |  | C5-C6 | 3 | 3 |
| 71 | M | 59 | C3-C4 | 4 | 3 |
|  |  |  | C4-C5 | 4 | 3 |
|  |  |  | C5-C6 | 5 | 4 |
| 72 | M | 49 | C3-C4 | 4 | 4 |
|  |  |  | C4-C5 | 4 | 4 |
|  |  |  | C5-C6 | 5 | 4 |
| 73 | F | 62 | C3-C4 | 4 | 4 |
|  |  |  | C4-C5 | 4 | 4 |
|  |  |  | C5-C6 | 5 | 3 |
| 74 | M | 48 | C3-C4 | 3 | 4 |
|  |  |  | C4-C5 | 3 | 4 |
|  |  |  | C5-C6 | 4 | 4 |
| 75 | F | 71 | C3-C4 | 4 | 3 |
|  |  |  | C4-C5 | 4 | 4 |
|  |  |  | C5-C6 | 5 | 4 |
| 76 | M | 45 | C3-C4 | 2 | 3 |
|  |  |  | C4-C5 | 3 | 3 |
|  |  |  | C5-C6 | 2 | 3 |
| 77 | F | 59 | C3-C4 | 4 | 3 |
|  |  |  | C4-C5 | 5 | 3 |
|  |  |  | C5-C6 | 5 | 3 |
| 78 | F | 47 | C3-C4 | 3 | 2 |
|  |  |  | C4-C5 | 4 | 3 |
|  |  |  | C5-C6 | 4 | 4 |
| 79 | M | 72 | C3-C4 | 4 | 3 |
|  |  |  | C4-C5 | 4 | 4 |
|  |  |  | C5-C6 | 4 | 4 |
| 80 | F | 71 | C3-C4 | 4 | 3 |
|  |  |  | C4-C5 | 5 | 3 |
|  |  |  | C5-C6 | 5 | 4 |
| 81 | M | 64 | C3-C4 | 4 | 3 |
|  |  |  | C4-C5 | 3 | 2 |
|  |  |  | C5-C6 | 3 | 3 |
| 82 | F | 38 | C3-C4 | 2 | 4 |
|  |  |  | C4-C5 | 3 | 4 |
|  |  |  | C5-C6 | 2 | 4 |
| 83 | M | 51 | C3-C4 | 3 | 2 |
|  |  |  | C4-C5 | 3 | 3 |
|  |  |  | C5-C6 | 4 | 2 |
| 84 | F | 56 | C3-C4 | 3 | 2 |
|  |  |  | C4-C5 | 4 | 2 |
|  |  |  | C5-C6 | 4 | 2 |
| 85 | M | 54 | C3-C4 | 2 | 3 |
|  |  |  | C4-C5 | 2 | 3 |
|  |  |  | C5-C6 | 3 | 4 |
| 86 | M | 41 | C3-C4 | 3 | 2 |
|  |  |  | C4-C5 | 3 | 3 |
|  |  |  | C5-C6 | 3 | 3 |
| 87 | M | 53 | C3-C4 | 3 | 4 |
|  |  |  | C4-C5 | 3 | 3 |
|  |  |  | C5-C6 | 2 | 3 |
| 88 | M | 60 | C3-C4 | 4 | 3 |
|  |  |  | C4-C5 | 3 | 4 |
|  |  |  | C5-C6 | 5 | 4 |
| 89 | M | 23 | C3-C4 | 2 | 2 |
|  |  |  | C4-C5 | 2 | 3 |
|  |  |  | C5-C6 | 3 | 3 |
| 90 | F | 49 | C3-C4 | 2 | 2 |
|  |  |  | C4-C5 | 2 | 3 |
|  |  |  | C5-C6 | 3 | 3 |
| 91 | M | 52 | C3-C4 | 2 | 4 |
|  |  |  | C4-C5 | 2 | 4 |
|  |  |  | C5-C6 | 3 | 4 |
| 92 | M | 46 | C3-C4 | 1 | 3 |
|  |  |  | C4-C5 | 2 | 3 |
|  |  |  | C5-C6 | 2 | 4 |
| 93 | M | 49 | C3-C4 | 4 | 4 |
|  |  |  | C4-C5 | 4 | 4 |
|  |  |  | C5-C6 | 5 | 3 |
| 94 | M | 65 | C3-C4 | 3 | 3 |
|  |  |  | C4-C5 | 3 | 3 |
|  |  |  | C5-C6 | 4 | 4 |
| 95 | F | 37 | C3-C4 | 3 | 4 |
|  |  |  | C4-C5 | 4 | 3 |
|  |  |  | C5-C6 | 3 | 3 |
| 96 | M | 31 | C3-C4 | 2 | 2 |
|  |  |  | C4-C5 | 2 | 3 |
|  |  |  | C5-C6 | 2 | 3 |
| 97 | F | 32 | C3-C4 | 2 | 2 |
|  |  |  | C4-C5 | 3 | 3 |
|  |  |  | C5-C6 | 2 | 2 |
| 98 | F | 41 | C3-C4 | 3 | 3 |
|  |  |  | C4-C5 | 3 | 3 |
|  |  |  | C5-C6 | 4 | 3 |
| 99 | M | 37 | C3-C4 | 4 | 3 |
|  |  |  | C4-C5 | 4 | 4 |
|  |  |  | C5-C6 | 3 | 4 |
| 100 | M | 35 | C3-C4 | 2 | 2 |
|  |  |  | C4-C5 | 2 | 3 |
|  |  |  | C5-C6 | 3 | 3 |
| 101 | M | 50 | C3-C4 | 2 | 2 |
|  |  |  | C4-C5 | 2 | 2 |
|  |  |  | C5-C6 | 3 | 3 |
| 102 | M | 56 | C3-C4 | 2 | 2 |
|  |  |  | C4-C5 | 3 | 3 |
|  |  |  | C5-C6 | 2 | 2 |
